# The effect of capacity building evidence-based medicine training on its implementation among healthcare professionals in Southwest Ethiopia: a controlled quasi-experimental outcome evaluation

**DOI:** 10.1186/s12911-023-02272-7

**Published:** 2023-08-31

**Authors:** Habtamu Setegn Ngusie, Mohammadjud Hasen Ahmed, Shegaw Anagaw Mengiste, Mihretu M. Kebede, Shuayib Shemsu, Shuma Gosha Kanfie, Sisay Yitayih Kassie, Mulugeta Hayelom Kalayou, Monika Knudsen Gullslett

**Affiliations:** 1 Department of Health Informatics, School of Public Health, College of Medicine and Health Sciences, Woldia University, Woldia, Ethiopia; 2 Department of Health Informatics, College of Health Sciences, Mettu University, Mettu, Ethiopia; 3 University of South-Eastern Norway, Post office box 235, N-3603 Kongsberg, Norway; 4 German Cancer Research Center (DKFZ), Heidelberg, Germany; 5 Department of Public Health, College of Health Sciences, Mettu University, Mettu, Ethiopia; 6 Department of Health Informatics, School of Public Health, College of Medicine and Health Sciences, Wollo University, Dessie, Ethiopia; 7 Faculty of Health and Social Sciences, University of South-Eastern Norway, Drammen, Norway

**Keywords:** Capacity building training, Competence, Evidence-based medicine, Evidence-based practice, Healthcare professionals

## Abstract

**Background:**

Evidence-based medicine (EBM) bridges research and clinical practice to enhance medical knowledge and improve patient care. However, clinical decisions in many African countries don’t base on the best available scientific evidence. Hence, this study aimed to determine the effect of training interventions on background knowledge and awareness of EBM sources, attitude, competence, and practice of EBM among healthcare professionals.

**Method:**

We designed a controlled group quasi-experimental pre-post test study to evaluate the effect of capacity-building EBM training. A total of 192 healthcare professionals were recruited in the study (96 from the intervention and 96 from the control group). We used a difference-in-differences (DID) analysis to determine the effect of the training. Along the way, we used a fixed effect panel-data regression model to assess variables that could affect healthcare professionals’ practice of EBM. The cut point to determine the significant effect of EBM training on healthcare professionals’ background knowledge and awareness of EBM sources, attitude, and competence was at a P-value < 0.05.

**Result:**

The DID estimator showed a significant net change of 8.0%, 17.1%, and 11.4% at *P* < 0.01 on attitude, competence, and practice of EBM, respectively, whereas no significant increment in the background knowledge and awareness of EBM sources. The fixed effect regression model showed that the attitude [OR = 2.288, 95% CI: (1.049, 4.989)], competence [OR = 4.174, 95% CI: 1.984, 8.780)], technical support [OR = 2.222, 95% CI: (1.043, 3.401)], and internet access [OR = 1.984, 95% CI: (1.073, 4.048)] were significantly affected EBM practice.

**Conclusion:**

The capacity-building training improved attitude, competence, and EBM practice. Policymakers, government, and other concerned bodies recommended focusing on a well-designed training strategy to enhance the attitude, competence, and practice towards EBM among healthcare professionals. It was also recommended to enhance internet access and set mechanisms to provide technical support at health facilities.

**Supplementary Information:**

The online version contains supplementary material available at 10.1186/s12911-023-02272-7.

## Background

Evidence-based decision-making is a strategic method of applying empirical knowledge and research findings [1, 2]. Evidence-based Decision-making at healthcare facilities has shown improvement over the past few decades due to the advancement of EBM [3, 4].EBM is a method for determining the safety and efficacy of medical therapies and public health interventions [5]. EBM avoids decisions based on gut feeling and enables it to rely on organized facts obtained from scientific literature, organizational data, professional expertise, values, and concerns of stakeholders [6].

Healthcare professionals should provide clinical services based on the best and rigorously tested evidence [7]. EBM is the ‘conscientious, explicit and judicious use of current best evidence in making decisions about individual patients’ [7, 8]. Despite this, the slow and haphazard process of promoting, translating, and implementing research findings into clinical compromises the potential benefits of clinical and public health research [9].

The practice of EBM involves the integration of three pillars such as up-to-date research evidence, clinical expertise, and predicaments, rights, and preferences of the patients in making clinical decisions about their care [10, 11]. We can summarize the concept of EBM into five-step models such as 1/ formulating answerable clinical questions, 2 / searching evidence, 3 / appraising the evidence, 4 / deciding to integrate evidence with your clinical expertise and the patient’s values, and 5/ evaluating the performance [12].

EBM has been used to guide policy formulation and implementation of preventive and curative interventions in Europe, North America, and Australia [13]. In this regard, around a hundred institutions have been established to synthesize research evidence, develop systematic reviews, policy briefings, and health technology assessments, and set guidelines for practice [14, 15]. Despite the aforementioned signs of progress, the European Centre for Disease Prevention and Control (ECDC) reports only 7% of the vaccination-based interventions achieved the highest grade evidence, which means clinical decisions with meta-analysis and randomized control trials [16]. In line with this, extant literature accentuated that most decisions in the world healthcare system are still done based on the low-quality evidence reported from case reports, case series, and observational studies [17].

When we come to Africa, many factors exacerbate the problems. Countries in this continent don’t always base their healthcare decisions on the best available scientific evidence [18–21]. A literature review signifies that talking about the level of evidence in the health systems of this continent was deemed luxurious. The inadequate background knowledge, competence, and behavior of the healthcare professionals are a challenge to implementing EBM in the continent [22, 23]. Healthcare professionals in Sub-Saharan Africa lack the competence to critically review and judge the quality of research [24]. The other problem that aggravated the situation is that only a little randomized research has been conducted for clinical conditions in this sub-continent [25, 26].

In Ethiopia, the Ministry of Health (MOH) has been working on building the healthcare human resource capacity by distributing clinical guidelines to all healthcare facilities [27, 28]. Nevertheless, those clinical guidelines haven’t been developed based on assessing high-grade evidence from systematic literature reviews and meta-analyses. Similar to other low-resource countries, clinical guidelines in Ethiopia are not timely updated to include the latest evidence from high-level medical research [29–31].

Additionally, teaching EBM is not yet a component of the undergraduate medical and health sciences curriculum, which results in a gap in background knowledge, behavior, competence, and practice of EBM [32]. Those situations lead most healthcare professionals to build their clinical decisions based on what they had learned from undergraduate or postgraduate classes; they didn’t use updated evidence [33, 34].

Previous studies revealed that 32.3–57.6% [27–29, 35–40] of healthcare professionals in Ethiopia integrate EBM into their medical practice. Few studies also determined background knowledge and awareness of EBM sources [27, 36, 41–47], attitude [27, 36, 39, 41, 43, 44, 46–48], and competence [49, 50] of healthcare professionals for EBM.

Literature also revealed that experience [51], age [52], educational level [53], workload or insufficient time [27, 36, 54, 55], sufficient hardware [55], poor internet access [27, 36], lack of technical support [54], knowledge of statistical terms [56], self-efficacy [52], lack of interest to find research reports [35, 39], and lack of patient cooperation [57, 58] were commonly depicted determinants of background knowledge and awareness of EBM sources, attitude, competence and practice of EBM.

Training has been the commonly recommended strategy to improve the background knowledge and awareness of EBM sources, attitude, competence, and practice of EBM. We argue that if we trained healthcare professionals to know the relevance of EBM, then it is more likely to integrate EBM into their daily medical practice and patient care. Previous studies reported training as one of the determinant factors of EBM [35, 36], but only a few studies examined its causal effect on healthcare professionals’ background knowledge and awareness of EBM sources, attitude, competence, and practice of EBM [59–62].

Examining the impact of training would enable us to identify the prospects to scale up the capacity of EBM. Therefore, we hypothesized that a well-designed training intervention could enhance background knowledge and awareness of EBM sources, attitude, competence, and practice.

## Methods and materials

### Study design and setting

We used a quasi-experimental study design using controlled before and after the study to evaluate the effect of capacity-building training on the implementation of EBM. A two-week capacity-building training from January 18 to February 2, 2022, was given to randomly selected healthcare professionals working at three hospitals in Ilu Ababa Bora and Buno Bedelle Zones, which were together called Ilubabor until recent times. Conversely, a controlled group was selected from two hospitals that didn’t take the training during the project period.

**Fig. 1:**
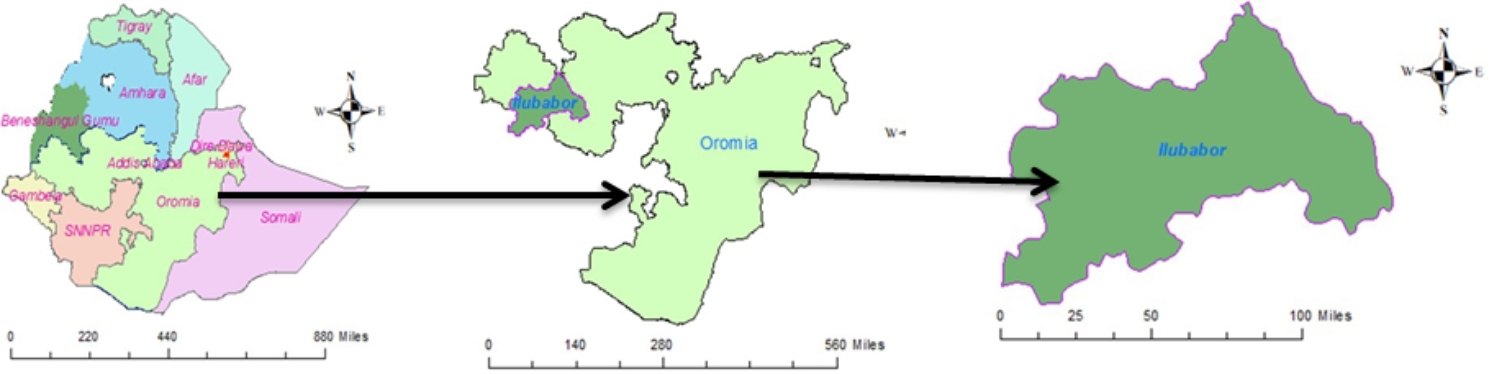
Map of the study area for Ilubabor, Oromia Region, Southwest Ethiopia

The measurement for both the intervention and control groups was taken at two points in time, in which the baseline data was taken, before the training, from January 10 to 17. End-line measurement for both groups was taken, after four months of the training days, from June 3 to 14, 2022. The control and the intervention groups were healthcare professionals in Ilu Aba Bora and Buno Bedele Zones, Oromia Region, Southwest Ethiopia (See Fig. 1).

### Study population

The source population of this study included all healthcare professionals working at public hospitals in Ilu Aba Bora and Buno Bedelle zones. Healthcare professionals who were on leave or retired were excluded from the study. Additionally, healthcare professionals who had previous exposure to EBM training, who had less than six months of experience, or who were not permanent employees were excluded from both the control and intervention groups of the study.

### Sample size and sampling technique

We used the comparison of two population proportions formulas in the baseline and end-line components of the study. We took the largest after calculating the sample size for the primary outcome variable (the practice of EBM) and secondary outcome variables (background knowledge and awareness of EBM sources, attitude, and competence). Accordingly, a 55% prevalence of EBM practice from the previous study [35] was taken. Additionally, the probability score at 95% intervals, and a precision level of 5%, were considered.

$${\text{n}} = ({({{\text{Z}}_1}_{ - a} + {{\text{Z}}_1}_{ - b})^2}[{\text{P}}1{\text{ }}(1-{\text{P}}1)\, + \,{\text{P}}2{\text{ }}(1-{\text{P}}2)\left] {)/(} \right[{\text{P}}1-{\text{P}}2]2)$$

Z1-α = 1.96, standard normal deviate corresponding to the 95% confidence interval.

Z1-β = 0.840, standard normal deviate corresponding to 80% power of the study.

p1 = 0.55, the proportion of the outcome variable by reviewing the previous studies, whereas p2 is the expected level of practice at the end of the project in which an increase of 20% in EBM practice was expected. Accordingly, we got n = 91. Then we added a 5% non-response rate, and the final sample size became 96 for each group.

There were five hospitals in Ilu Aba Bora and Buno Bedelle zones. The total sample size obtained based on the above calculation (96) was proportionally allocated to three hospitals that were planned to be addressed in the capacity-building training. Then, healthcare professionals for the intervention group were selected with a simple random sampling technique from each hospital.

On the other hand, the total sample size obtained based on the above calculation (96) was proportionally allocated to two hospitals that weren’t addressed by the training intervention. Then, healthcare professionals for the control group were selected with a simple random sampling technique from each hospital.

The selections of those three hospitals for the intervention group were purposively based on the direction given by the Mettu University Office of community service. This was because the three hospitals were located at the community service site of Mettu University. The left two hospitals found in a similar zone but not in the community service site were considered control hospitals. The sampling frame was prepared based on human resource healthcare professionals’ profiles and the study objective in each hospital to select the participants.

### Intervention

We designed a capacity-building training project, which aimed at enhancing the implementation of EBM by improving the background knowledge and awareness of EBM sources, attitude, competence, and practice of healthcare professionals at public health facilities in southwest Ethiopia. The training content and aim were evaluated by Mettu University community service coordinators and experts in the field.

Following the completion of the baseline data collection, training on EBM was given for a total of 2 weeks, from January 18 to February 2, 2022. The end-line survey was collected four months after the baseline survey (from June 3 to 14, 2022). The training was given only to the intervention groups. It took a total time of 41 h (8 h of discussion, 11 h of lectures, and 22 h of practical or EBM labs). The mode of delivery was face-to-face. The lab session was delivered at three digital labs of Mettu University. Trainees were guided by trainers during lab sessions. A discussion was planned to be at the end of lectures in which the trainer’s role was as a facilitator.

An EBM module was developed by reviewing different literature [9, 19, 22, 61, 63–67] and Johns Hopkins Nursing Evidence-Based Practice (JHNEBP) Model [68]. The module was distributed to trainers via soft or hard copy based on preference. Six health informatics and public health experts were involved in developing and reviewing the module as well as to deliver the training. The two trainers have master’s degrees in health informatics; the left 4 are an assistant professors and above in public health and health informatics.

The training module was classified into nine lessons. The training module mainly focused on the introduction and principles of EBM, formulating a focused clinical question, finding the current best evidence, evaluating the quality of the evidence, using an online database like PubMed and Cochrane Library, searching strategy, critical appraisal, interpreting research results, diagnostic test, validity appraisal, systematic review, meta-analysis and so on (See **Additional file 2**). A syllabus containing the detail of the training content was given before the commencement of the training. Case scenarios prepared by trainers were given to trainees during their practical sessions.

### Outcome measure

The primary outcome variable of this study was the effect of capacity-building training on the EBM practice of healthcare professionals. The secondary outcomes variable included the change in background knowledge and awareness of EBM sources, attitude, and competence of EBM.

### Operational definition

#### Healthcare professionals

For this study, healthcare professionals were operationalized as employees of health facilities who had at least a diploma certificate in the health profession and provide clinical care for patients or clients [69].

#### Workload

Healthcare professionals who worked more than 8 h per day were considered as having a workload or coded as ‘yes’ [70].

### Instrument

The researchers developed questionnaires that met the study’s purpose. The items of the questionnaires focused on the healthcare professionals’ background knowledge and awareness of EBM sources, attitude, competence, and practice of EBM, and other individual factors such as age, sex, education level, and so on. The data collection tool was prepared in the English language. The instrument was assessed for content validity. A pre-test was done in Jimma referral hospital, which was not part of the actual data collection but has a similar study setting. Accordingly, the required corrections in language and content were done before the actual data collection commenced. Data was gathered by a self-administered questionnaire. **Background knowledge and awareness of EBM sources**: It was measured by 14 items with two response categories (1 = Yes and 2 = No). Finally, the responses were dichotomized into two “0” and “1”. If respondents responded as yes, it was recoded as “1” otherwise it was recoded as “0”. The normality test for background knowledge and awareness of EBM sources showed skewed distribution, so we computed the median, and healthcare professionals who responded correctly equal to or above the median were labeled as having a good background knowledge and awareness of EBM sources. On the other hand, healthcare professionals who responded below the median were considered as having poor background knowledge and awareness of EBM sources.

#### Attitude toward EBM

In implementation science, an attitude would be described as how positively one is predisposed towards using a particular evidence-based practice (EBP) [71]. The tool used to measure the attitude consisted of 11 Likert scale items ranging from 1 or strongly disagree to 5 or strongly agree. It was dichotomized into two favorable and unfavorable attitude based on the mean value.

#### Competence of EBM

Even though there are different commonly used EBM test tools to measure competence, such as the Fresno test [72] and Berlin test [64], we chose to use the assessing competence in EBM (ACE) tool [73]. The ACE tool is more comprehensive as it incorporates all EBM steps. The ACE tool also permits alternate scenarios to control the potential impact of recall bias during testing. The tool has a ‘yes’ or ‘no’ binary format, which could be answered based on provided patient case scenario. We used different scenarios for the pretest and posttest.

The total items of the questioner were 15, which mainly focuses on the five-step model of EBM [12], such as formulating answerable clinical questions (2 items), finding the evidence (2 items), critically appraising the evidence for its validity and usefulness (7 items), and applying the evidence and evaluating its performance (3 items). A high score means good competence in finding evidence for decisions in their daily practices.

#### EBM practice

It refers to the use of EBM in healthcare institutions. It was measured by 11 Likert scale questions ranging from 1 = never, 2 = rarely, 3 = sometimes, 4 = most of the time, and 5 = always. All individual answers to practice questions were computed to obtain total mean scores and categorized as good practice (if participants scored ≥ mean score) or poor practice (if participants scored < mean score) (See **Additional file 3**).

### Statistical analysis

We used Epi-Data version 4.6 and STATA version 14.0 for data entry and analysis, respectively. Descriptive data were used to describe the demographic characteristics of participants. The baseline differences in the socio-demographic variables between the intervention and controlled groups were tested using either an independent-samples t-test for continuous variables or a chi-square for nominal/categorical variables.

To assess the effect of capacity-building community service training on the primary and secondary outcomes, DID analysis using a 5% level of significance was used. Our data was panel data set or repeated measurement of one variable a minimum of two times. Therefore, we used the Hausman test to select which model was appropriate to analyze our data among different models applicable for panel data analysis. Finally, the test showed that the fixed effect regression model was appropriate to study the effect of other variables on the primary outcome variable.

Both the adjusted R-squared (R2 = 0.736) and the F-test (F-statistic = 43.72, p < 0.001) showed that the fixed effect model was a fitted panel model for our data set and had high explanatory power than other panel models. According to its assumption, the fixed effect removes the effect of time-invariant characteristics (e.g. sex, age, and so on); thereby it controls type II error. This model assesses the net effect of the time-varying predictors on the outcome variable. The odds ratio (OR) with the corresponding 95% confidence intervals was reported to show the strength of the association. A P-value < 0.05 was considered as a cut point to indicate statistical significance in all analyses.

## Result

### Characteristics of study participants

The study comprised 192 healthcare professionals, with 96 for the intervention group and 96 of them for the control group. Among the total participants approached in the intervention group, 89(92.7%) and 83(86.5%) of them gave a response at baseline and the completion of the study, respectively. All 96(100%) eligible study subjects of the control group completed the self-administered questionnaires in the pre-intervention whereas only 87(90.6%) of this group gave a response in the post-intervention periods.

In addition, when the pretest of each group was analyzed, 59.2% of the intervention group and 71.6% of the control group participants were in the age group below 30 years. As the pretest data showed, males constituted 53.9% of the intervention group and 63.5% of the control group. In addition, among the intervention group study participants in the pretest period, the majority, 55.1% were nurse professionals. Whereas, among those participants in the control group during the pretest period and those nurse professionals were 38.5%. Details of individual characteristics of the study participants at baseline in both groups are reported in Table 1.

**Table 1:** Individual characteristics of healthcare professionals at baseline in Southwest Ethiopia, 2022

Demographic variables		Control group	Intervention group		Test statistics
		**Pre intervention n = 96(%)**	**Pre intervention n = 89(%)**		
Sex	Male	61(63.5)	48(53.9)	χ2 = 0.632, p = 0.802	
	Female	35(36.5)	41(46.1)		
Age[M (SD)]	**≤** 30	33(8.3)	28(6.7)	t = 1.652, p = 0.096	
Profession	Nurse	37(38.5)	49(55.1)	χ2 = 0.137, p = 0.890	
	Physician	23(24.0)	12(13.5)		
	HO	19(19.8)	22(24.7)		
	Others	17(17.7)	6(6.7)		
Educational level	Diploma	26(27.1)	17(19.1)	χ2 = 18.615, p = 0.03	
	Degree	59(61.5)	66(74.2)		
	Master	11(11.4)	6(6.7)		
Experience	**≤5 years**	38(39.6)	22(24.7)	χ2 = 0.033, p = 0.891	
	**5–10 years**	51(53.1)	56(62.9)		
	**> 10 years**	7(7.3)	11(12.4)		
Monthly Salary	**≤** 5,000 ETB	33(34.4)	21(23.6)	χ2 = 1.629, p = 0.730	
	> 5,000 ETB	63(65.6)	68(76.4)		
Workload	Yes	59(61.5)	57(64.0)	χ2 = 0.95, p = 0.26	
	No	37(38.5)	32(36.0)		
Technical support	Yes	9(9.4)	16(18.0)	χ2 = 3.93, p = 0.032	
	No	87(90.6)	65(82.0)		
Having internet access	Yes	42(43.8)	37(41.6)	χ2 = 0.317, p = 0.067	
	No	53(56.2)	52(58.4)		
Having a computer in their office	Yes	45(46.9)	28(31.5)	χ2 = 0.106, p = 0.078	
	No	51(53.1)	61(68.5)		

### Effects of the capacity-building training intervention on primary and secondary outcome variables

The level of EBM practice for the control group was 43.8% (95% CI 38.6%-47.2%) at baseline and 46.0% (95% CI 39.9%-51.5%) at the study endpoint. The level of EBM practice for the intervention group was 38.2% (95% CI 34.8%-43.1%) at baseline and 51.8% (95% CI 46.6%-54.1%) at the study endpoint. The DID analysis indicated that the intervention resulted in an 11.4% net increment in the level of EBM practice among the intervention group compared to the control group. This increment was significant at a *P*-value < 0.01.

The proportion of background knowledge and awareness of EBM sources among healthcare professionals increased by 7.7% (from 56.2 to 63.9%) in the intervention group while it increased by 2.6% (from 58.3 to 60.9%) in the control group. The average proportion of background knowledge and awareness of EBM sources net difference between the two groups was 5.1%.

The proportion of attitude toward EBM among healthcare professionals increased by 14.5% (from 67.4 to 81.9%) in the intervention group while it increased by 6.5% (from 62.5 to 69.0%) in the control group. The average proportion of attitude net difference between the two groups was 8.0%.

Additionally, the proportion of competence of EBM among healthcare professionals increased by 22.4% (from 42.7 to 65.1%) in the intervention group while it increased by 5.3% (from 51.0 to 56.3%) in the control group. The average proportion of competence net difference between the two groups was 17.1%. The DID analysis showed the effect of the intervention was significant for attitude (*P*-value < 0.01) and competence (P-value < 0.001), whereas the net effect on background knowledge and awareness of EBM sources wasn’t significant (See Table 2 for more detail).

**Table 2:** DID analysis of background knowledge and awareness of EBM sources, attitude, competence, and practices of EBM between intervention and control groups during baseline and end-line surveys (n1 = n2 = 96)

Variables	Intervention group			Control group			DID
	**Baseline**	**End line**	**Difference (EL-BL)**	**Baseline**	**Follow up**	**Difference (EL-BL)**	
Background Knowledge and awareness of EBM sources	56.2%	63.9%	7.7%*	58.3%	60.9%	2.6%	5.1%
Attitude of EBM	67.4%	81.9%	14.5%***	62.5%	69.0%	6.5%	8.0%**
Competence	42.7%	65.1%	22.4%***	51.0%	56.3%	5.3%	17.1%***
Practice of EBM	38.2%	51.8%	14.2%***	43.8%	46.0%	2.2%	11.4%**

NB: *P < 0.05, **P < 0.01, ***P < 0.001, EL = End Line, BL = Base Line, DID = Difference in difference, EBM = Evidence based medicine

### Factors affecting EBM practice among healthcare professionals

To identify independent factors associated with the end-line-baseline difference of the differences in the mean EBM practice scores, we employed a fixed effect regression model. Variables included in the model were: sex, age, profession, educational level, experience, monthly salary, religion, workload, technical support, internet access, computer access, background knowledge and awareness of EBM sources, attitude, and competence of EBM.

The fixed effect was designed to study the cause of the change in practice within participants and time-varying characteristics deemed not to cause the change. Therefore, those time-invariant variables or variables that change at a constant rate over time were omitted. Finally, the model showed that attitude [OR = 2.288, 95% CI: (1.049, 4.989)], competence [OR = 4.174, 95% CI: 1.984, 8.780)], technical support [OR = 2.222, 95% CI: (1.043, 3.401)], and internet access [OR = 1.984, 95% CI: (1.073, 4.048)] showed a significant effect on EBM practice (See Table 3 for more detail).

**Table 3:** Fixed effect panel data modeling for independent predictors of EBM practice among healthcare professionals in Southwest Ethiopia, 2022

Predictors	OR	SE	Z	P>|z|		95% Confidence Interval	
						**Lower**	**upper**
Workload	0.931	0.322	0.21	0.835		0.472	1.834
Attitude	2.288	0.909	2.08	0.037		1.049	4.989
Competence	4.174	1.584	3.77	0.000		1.984	8.780
Background Knowledge and awareness of EBM sources	1.206	0.501	0.45	0.652		0.534	2.720
Technical support	2.222	0.602	3.69	0.000		1.043	3.401
Having a computer in the office	1.168	0.421	0.43	0.666		0.577	2.367
Internet access	1.984	0.706	2.17	0.030		1.073	4.048

NB: P>|z| or P-value < 0.05 was considered as a cut point for level of significance

## Discussion

The main aim of this quasi-experimental evaluation was to investigate the effect of capacity-building training intervention on background knowledge and awareness of EBM sources, attitude, competence, and practice of EBM among healthcare professionals.

There was no significant difference in background knowledge and awareness of EBM sources, attitude, competence, and practice of EBM between the intervention and control groups at the start of the study. However, after the training intervention, there was a significant difference between the intervention and control groups for attitude, competence, and practice of EBM.

Improvements in background knowledge and awareness of EBM sources were also found, but the change from the DID analysis was not significant. The insignificant result in background knowledge and awareness of EBM sources might be due to the training content not being focused on it. Additionally, knowledge gained from the training might be lost after some point in time if not reinforced by practical follow-ups or assessments.

This finding was in line with a study of Monash University students, which reported a significant effect of learning on attitude and practice [62]. However, our finding contradicts the study of Monash, which reported an insignificant effect on the competence of EBM. This contradiction could be due to the variation in the teaching strategies and the study participants. The study participants in Monash were medical students, which might not enhance their competence by day-to-day practice, unlike healthcare professionals. The content of the course in our study wasn’t similar to the study of Monash, which might be another possible justification for this variation.

An interventional study conducted in China is similar to the current study in that they have a significant effect of EBM training courses on primary healthcare professionals’ attitude [61]. However, it contradicts our finding that reported a significant effect of the training intervention on background knowledge and awareness of EBM sources.

The discrepancy might be due to the difference in the dosage of the intervention and training content. The intervention study from China provided eight weeks of training with two hours every day, but our intervention was only for two weeks. Additionally, the delivery of the training in China was accompanied by conferences. But, the study in China did not assess the effect of the intervention on EBM competence and practice.

Our finding also differs from the study findings reported from South Africa, which reported a significant effect of an education intervention on Knowledge; but an insignificant effect on attitude towards EBM [21]. This variation could be due to the differences in the contents of the training and tools used to measure the attitude and background knowledge and awareness of EBM sources. On the other hand, the interval time between the pre-test and post-test in the previous study was three months, whereas it was four months in the current study.

Accordingly, the long duration between pre and post-training may cause the loss of newly acquired knowledge from the intervention if it isn’t reinforced by practical follow-ups or supportive supervision, which might be a possible justification for this discrepancy. However, further study is required to confirm this and to determine the optimal duration of an effective EBM educational intervention.

In line with our study findings, the study in Taiwan [59] and Portugal [60] reported a significant positive effect of educational intervention on the behavior and competence of EBM. However, unlike our study findings, background knowledge and awareness of EBM sources showed a significant improvement after training. The discrepancy might be due to the study in Taiwan, and Portugal didn’t include a controlled group. Additionally, the study participants in the study of Taiwan, and Portugal were medical students.

The fixed effect model in this study implied that attitude is a significant predictor of the EBM practice. Healthcare professionals who had a favorable attitude were highly practicing EBM. The finding was corresponding with previous studies conducted elsewhere in the World [37, 40, 54]. It could be justified as the beliefs of individuals toward using the EBM is necessary versus unnecessary, beneficial versus harmful predispose them to apply in clinical practice.

The study found that competence was a significant factor in EBM practice, in which healthcare professionals with high competence levels were more likely to practice EBM than their counterparts. In agreement with our finding, a study in Northwest Ethiopia implied that the ability to apprise evidence was a significant factor of EBM practice [40], and also it was consistent with the study of central Ethiopia [38].

The possible justification for this finding could be competent healthcare professionals can do EBM tasks effectively and efficiently. It is impossible to find the best available evidence to apply in clinical practice without the competence of formulating a clinical question, finding information or evidence, critically appraising the information/evidence, integrating appraised evidence with clinical expertise and patient preferences, and evaluating the evidence.

This study showed that healthcare professionals who got technical support are more likely to practice EBM. This finding was supported by a study in Northwest Ethiopia [37, 38]. The possible justification for this could be due to technical support paving the doorway to obtaining guidance from experienced professionals in the field. Providing such support for healthcare professionals could enable them to ask how to access, appraise, apply, and audit evidence.

The result of this study confirms that internet access was one of the predictors of EBM practice. This finding was in agreement with previous reports [35, 36]. The justification for this could be that internet-enabled individuals access sources of information or evidence from digital platforms.

### Strengths and limitations of the study

The issue of omitted variable bias could also reduce due to the fixed effect regression model. However, it didn’t conclude causal associations between an intervention and an outcome since randomization couldn’t use in a quasi-experimental study. The data was collected based on self-reported information; this might cause an overestimation of participants’ practice. We relabeled a few variables after data collection because they needed modification to make the labeling representative of each item in the tool. This might be after participants’ responses were already affected due to confusion about its labeling (e.g. knowledge was relabeled to background knowledge and awareness of EBM sources).

The effect of the training was determined based on statistical significance, which might not represent public health or clinical significance. In this study, the baseline data was collected before the training. The end-line measurement was also taken after four months of the training, whereas the immediate and intermediate effect of the training wasn’t assessed. We based the statistical analysis plan only on practices of EBM, which not addressed the possible factors of background knowledge and awareness of EBM sources, attitude, and competence.

## Conclusions

The capacity-building training improved attitude, competence, and EBM practice. Policymakers, government, and other concerned bodies recommended focusing on a well-designed training strategy to enhance the attitude, competence, and practice towards EBM among healthcare professionals. It was also recommended to enhance internet access and set mechanisms to provide technical support at health facilities.

### Electronic supplementary material

Below is the link to the electronic supplementary material.

**Additional file 1**. GREET 2023 checklist for EBM training in Southwest Ethiopia, based upon the TIDieR guidance

**Additional file 2.** Supportive Table 1: EBM module and leaning objectives to providing training for healthcare professionals in southwest Ethiopia, 2022: developed based on different models and literatures.

**Additional file 3.** Appendix A: Data collection tool.

## Data Availability

Data supporting this study are not publicly available due to legal or ethical restrictions on sharing the data publicly. However, data are available upon a reasonable request from the corresponding author.

## Abbreviations

**OR**: Odds Ratio
**ACE**: Assessing Competence in Evidence
**CI**: Confidence Intervals
**DID**: Difference in Difference
**EBM**: Evidence-Based Medicine
**EBP**: Evidence-Based Practice
**ECDC**: European Centre for Disease Prevention and Control
**JHNEBP**: Johns Hopkins Nursing Evidence-Based Practice
**MeSH**: Medical Subject Headings
**MOH**: Ministry of Health
**Ph.D.**: Doctor of Philosophy
**SE**: Standard Error
**SD**: Standard Deviation
