# Supplementary material for: The effect of capacity building evidence-based medicine training on its implementation among healthcare professionals in Southwest Ethiopia: a controlled quasi-experimental outcome evaluation
Source: BMC Med Inform Decis Mak. 2023 Aug 31;23:172. doi: 10.1186/s12911-023-02272-7 (PMC10472735; doi:10.1186/s12911-023-02272-7)
Supplement: Supplementary file 1 — Additional file 1. GREET 2023 checklist for EBM training in Southwest Ethiopia, based upon the TIDieR guidance [file 12911_2023_2272_MOESM1_ESM.pdf]

Additional file 1: GREET 2023 checklist for EBM training in Southwest Ethiopia, based upon the TIDieR guidance[1]

BRIEF NAME
**1. INTERVENTION:** A quasi-experimental study design using controlled before and after the study to evaluate the effect of capacity-building training on the implementation of EBM was used. A two-week capacity-building training from January 18 to February 2, 2022, was given to randomly selected healthcare professionals. Conversely, a controlled group was selected from two hospitals that didn't have the training during the project period. The training consisted of computer lab training on EBM, lectures, and group discussions. The training module mainly focused on the introduction and principles of EBM, formulating a focused clinical question, finding the current best evidence, evaluating the quality of the evidence, using an online database like PubMed and Cochrane Library, searching strategy, critical appraisal, interpreting research results, diagnostic test, validity appraisal, systematic review, and meta-analysis and so on.
**WHY - this educational process**
2. **THEORY: THEORY:** This training intervention was designed by public health and health informatics experts based on reviewing literature and extent models in EBM practice such as Johns Hopkins Nursing Evidence-Based Practice (JHNEBP) Model[2], the Five-Step Model of EBP[3] and The Four Component Instructional Design Model (4C/ID)[4]. We used a quasi-experimental outcome evaluation to ascertain the effectiveness of EBM training on the attitude, competence, and practice of healthcare professionals.<br>3. LEARNING OBJECTIVES: The main objective was to evaluate the effectiveness of capacity-building EBM training on the attitude, competence, and practice of EBM.<br>Upon completion of the training, trainees be able to:<br>- Understand the basic concepts of evidence-based medicine.

[1] Hoffmann TC, Glasziou PP, Boutron I, et al. Better reporting of interventions: template for intervention description and replication (TIDieR) checklist and guide. BMJ 2014; 348: g1687.

[2] Hunt JJNM. Johns Hopkins nursing evidence-based practice. ProQuest. 2012;19(7):8.

[3] Dawes M, Summerskill W, Glasziou P, Cartabellotta A, Martin J, Hopayian K, Porszolt F, Burls A, Osborne J and Second International Conference of Evidence-Based Health Care Teachers and Developers 2005: Sicily statement on evidence-based practice. BMC Med Educ 2005, 5(1):1.

[4] Maggio LA, Cate Ot, Irby DM, O'Brien BC. Designing evidence-based medicine training to optimize the transfer of skills from the classroom to clinical practice: applying the four component instructional design model. Academic Medicine. 2015;90(11):1457-61.

- Asking answerable clinical questions
- Appraising the evidence
- Creating a PICO Question

The detailed attributes are reflected in the learning contents and objectives, listed in **Supplementary file 2**.

4. EBP CONTENT: The EBM training module was designed based on published studies and extant EBM models. The module had nine lessons namely: introduction to evidence-based medicine, formulating clinical questions, finding the current best evidence, critical appraisal questions, using online sources for EBM, searching and summarizing the scientific literature, introduction to systematic review and meta-analysis, understanding research and level of evidence, and EBM in diagnosis, therapy, prognosis, and harm.

WHAT

5. MATERIALS: A syllabus containing the detail of the training content was given before the commencement of the training. The training module was provided to all trainees. Case scenarios prepared by trainers were given to trainees during their practical sessions. The control group did not attend the training or receive any materials from Books from an online source. The website to find the books is found in the footnote .

6. EDUCATIONAL STRATEGIES: The training was face-to-face which incorporated lectures, discussions, and exercises or digital skill lab practices on EBM.

7. INCENTIVES: There were no financial incentives except the per-diem for the trainees during the training period.

WHO PROVIDED

8. INSTRUCTORS: Six health informatics and public health experts were involved in delivering the training. The two trainers have master's degrees in health informatics; the left 4 are assistant professors and above in public health and health informatics. All trainers have a minimum of 4 years of research and teaching experience in EBM, research methodology, and medical informatics.

HOW

9. DELIVERY: The training was delivered thru face to face mode of delivery which incorporated lectures, discussions, and exercises or digital skill lab practices on EBM. The training was delivered at three digital labs of Mettu University. A total of 96 health care professionals were planned to be distributed to three digital labs (within three groups). However, 7 health care professionals weren't attending the training. There were six instructors which made the ratio of trainers to trainees 1:16.

WHERE

10. ENVIRONMENT: The trainees were selected from hospitals in southwest Ethiopia. The minimum

and maximum educational level of the trainees was diploma and master degree respectively. The majority of them (74.2%) were first-degree holders. The EBM training was 40 hours in duration and was delivered in three computer labs at Mettu University.
**WHEN and HOW MUCH**
**11. SCHEDULE:** The training was given for two-week from January 18 to February 2, 2022. The intervention group took the training from Monday to Saturday for 3:25 per day (105 minutes in the morning and 100 minutes in the afternoon). Breakfast, snacks, and lunchtime were considered on each training day. The training day did not include Sunday. Accordingly, the intervention group took the training for a total of 41 hours (For 12 days which is 3:25 per day).<br>12. Total contact time within a day was 3:25 (105 minutes in the morning and 100 minutes in the afternoon). The trainer gave a lecture and instruction for 55 minutes per day followed by a 40-minute discussion and 110 minutes for a lab session.
**PLANNED CHANGES**
13. The trainer encouraged reading materials given with soft copy or hard copy but it was up to them. A practical session was given with the guidance of the trainer.
**UNPLANNED CHANGES**
14. Technical assistance helped to solve technical issues like installing browsers, correcting corrupted computers, and network errors. The discussion was planned to be at the end of lectures but few of those undertook after the practice.
**HOW WELL**
**15. ATTENDANCE:** All participants who availed at the commencement of the training were present in all session of the training.
**16. Fidelity:** The teaching and learning strategies used in the educational intervention were delivered as planned except additional case scenario prepared for discussion.
17. The number of sessions, frequency, timing and duration for the educational intervention were delivered as scheduled.
