# Supplementary material for: The effect of capacity building evidence-based medicine training on its implementation among healthcare professionals in Southwest Ethiopia: a controlled quasi-experimental outcome evaluation
Source: BMC Med Inform Decis Mak. 2023 Aug 31;23:172. doi: 10.1186/s12911-023-02272-7 (PMC10472735; doi:10.1186/s12911-023-02272-7)
Supplement: Supplementary file 2 — Additional file 2. Supportive Table 1: EBM module and leaning objectives to providing training for healthcare professionals in southwest Ethiopia, 2022: developed based on different models and literatures. [file 12911_2023_2272_MOESM2_ESM.docx]

Supportive Table 1: EBM module and leaning objectives to providing training for healthcare professionals in southwest Ethiopia, 2022: developed based on different models and literatures^[[1]](#footnote-1),^^[[2]](#footnote-2)^

| **Modules** | **Learning Objectives** |
| --- | --- |
| Lesson one: Introduction to Evidence-Based Medicine | 1. Understand the basic concepts of evidence-based medicine. 2. Introduce goals and principles of EBM 3. Identify type of evidence 4. Understanding challenges of implementing EBM in health facilities |
| Lesson two: Formulating clinical questions | 1. Asking answerable clinical questions 2. Acquiring evidence 3. Appraising the evidence 4. Formulating a well-structured question 5. Creating a PICO Question 6. Identify components of a clinical question 7. Developing different types clinical questions |
| Lesson three: Finding the current best evidence | 1. Comparing source of evidence by setting the required criteria 2. Understand how to select up to date evidence 3. Understanding how to accesses evidence based tools 4. Understanding hierarchy of source of evidence 5. Identify print and online source of evidence |
| Lesson four: Critical Appraisal Questions | 1. Understand how to critically appraise questions in different health care setting 2. Understand techniques for ensuring validity and reliability of evidence before using it |
| Lesson five: Using Online Sources for Evidence Based Medicine | - 1. Develop skills to use online sources   2. Identify online sources   3. Understand advanced evidence searching techniques   4. Able to evaluate online sources |
| Lesson six: Searching and summarizing scientific literature | 1. Searching the Literature on Cochrane library and PubMed 2. Understand the process used in systematic reviews and identify a key source. 3. Use recommended guidelines for searching the scientific literature. |
| Lesson seven: Introduction to systematic review and meta-analysis | 1. Differentiate systematic review and Meta-analysis 2. Understanding reports of systematic review and Meta-analysis 3. Understanding and practicing steps to conduct systematic review and meta-analysis |
| Lesson eight: Understanding research and level of evidence | 1. Understanding basic concepts of research 2. Formulating the research problem 3. Formulating research questions, hypotheses, and objectives 4. Understanding bias and level of evidence 5. Able to interpret research result 6. Be able to use economic evaluation studies to justify, prioritize, and implement prevention and treatment strategies |
| Lesson nine: EBM in diagnosis, therapy, prognosis, and harm | 1. Identifying types of reports on diagnosis and therapy 2. Knowing types of reports in harm/etiology 3. Identifying types of reports on prognosis 4. Practicing evidence-based medicine in real time using diagnosis, therapy, prognosis, and harm questions 5. Understanding accuracy and validity of evidence 6. Enabling validity appraisal |

1. Maggio, Lauren A., et al. "Designing evidence-based medicine training to optimize the transfer of skills from the classroom to clinical practice: applying the four component instructional design model." Academic Medicine 90.11 (2015): 1457-1461. [↑](#footnote-ref-1)
2. Hunt, Jennifer. "Johns Hopkins nursing evidence-based practice." Nursing Management (through 2013) 19.7 (2012): 8. [↑](#footnote-ref-2)
