# Supplementary material for: The effect of capacity building evidence-based medicine training on its implementation among healthcare professionals in Southwest Ethiopia: a controlled quasi-experimental outcome evaluation
Source: BMC Med Inform Decis Mak. 2023 Aug 31;23:172. doi: 10.1186/s12911-023-02272-7 (PMC10472735; doi:10.1186/s12911-023-02272-7)
Supplement: Supplementary file 3 — Additional file 3. Appendix A: Data collection tool. [file 12911_2023_2272_MOESM3_ESM.docx]

# Appendix A: Data collection tool

**Part I: Individual characteristics of respondents**

**Instruction:** Below there are questions which intended to assess your background. Please circle the number in front of the option you choose or if you are asked to write a response, please do in the blank space provided.

| Se.no | Questions | Answer |
| --- | --- | --- |
| 101. | Age in Years | ­­­­____________ |
| 102 | Sex | 1.Male  2.Female |
| 103 | What are your current educational qualifications? | 1.Diploma nurse  2.BSC nurse  3.Diploma midwifery  4.BSC midwifery  5.General practitioner  6. Specialist physician  7. Other (specify)……….. |
| 104 | How long is your working experience (in years, or months)? | _____________________ |
| 105 | Profession | 1. Nurse  2. Midwife  3. Physician  4. HO  4. other specify___ |
| 106 | How money hours do you work per day? | __________________ |
| 107 | Monthly salary in ETB | _____________________ |
| 108 | Have you a computer in your office | 1. Yes 2. No |
| 109 | Is thee internet access in your health facilities | 1. Yes 2. No |
| 110 | Is there any technical support in your organization | 1. Yes 2. No |

**Part II: Background Knowledge and Awareness of EBM sources**

**Instruction:** Below are questions intended to assess your knowledge about evidence based practice. Please read and answer accordingly.

| **s.no** | **Question** | **Response** | **skip** |
| --- | --- | --- | --- |
| 201 | Have you heard of the term evidence-based practice or related terms? | 1. Yes 2. No | If no skip to Q 203 |
| 202 | If your response to the question above is ‘Yes’, what is your source of information? (more than one answer is possible) | 1. During my college or university education 2. From my colleague 3. I took training about evidence based practice 4. Other (specify)……… |  |
| 203 | Can you identify a site where clinical related journals, articles and guide lines published? | 1. Yes 2. No | If no skip to Q 203 |
| 204 | If yes for q 203 From where you found clinical information when you need to update yourself?(more than one answer is possible) | 1. PubMed 2. text books 3. Medscape 4. CINAHL 5. HINARY 6. UpToDate 7. Other specify_______ |  |
| 205 | Do you face difﬁcultly understanding research reports? | 1. Yes 2. No | If no skip to Q 207 |
| 206 | If yes for Q205 what is the reason? (more than one answer is possible) | 1. Difficulty understanding statistical data 2. Difficulty understanding epidemiological terms 3. Language problem 4. Other specify________ |  |
| 207 | Do you know the steps for evidence based practice? | 1. Yes 2. No | If no skip to Q 209 |
| 208 | If yes, how many steps are there for evidence based practice? | 1. 3 2. 4 3. 5 4. Other (specify)____ |  |
| 209 | Do you know the implications of research information for your clinical practice? | 1. Yes 2. No | If no skip to Q 211 |
| 210 | If yes for Q 210 what is the importance? (more than one answer is possible) | 1. It can improve patient outcomes 2. It increases quality of care 3. I can learn about recent, and best types of patient care 4. Others specify________ |  |
| 211 | Do you know how to implement evidence-based practice sufﬁciently to make practice changes? | 1. Yes 2. No |  |

**Part III: Questions related to professionals’ attitudes towards EBM**

**Instruction:** Below are questions intended to assess your attitude towards evidence based practice. Please circle one number for each question that describes your attitude best.

| S.N o | Questions | Strongly Agree | Agree | Neither agree nor disagree | Disagree | Strongly Disagree |
| --- | --- | --- | --- | --- | --- | --- |
| 301 | Implementing evidence based practice will improve the care that I deliver to my patients. | 0 | 1 | 2 | 3 | 4 |
| 302 | Evidence-based practice is not relevant to my profession. | 0 | 1 | 2 | 3 | 4 |
| 303 | Critically appraising evidence is an important step in the evidence based practice process. | 0 | 1 | 2 | 3 | 4 |
| 304 | Training should be given about evidence based practice. | 0 | 1 | 2 | 3 | 4 |
| 305 | Recent national guidelines can improve clinical care. | 0 | 1 | 2 | 3 | 4 |
| 306 | Evidence based practice takes too much time so it is difficult to implement | 0 | 1 | 2 | 3 | 4 |

**Part IV: Questions regarding to level of EBM practice**

**Instruction:** Below there are 19 questions about evidence-based medicine practice. Please answer each question by selecting the number that best describes how often each item has been done by you in the past 2 months. 1=never, 2= rarely, 3= sometimes, 4=most of the time, and 5=always

|  | **In the past 2 months, how often you** | |  |  |  |  |  |
| --- | --- | --- | --- | --- | --- | --- | --- |
| 401 | Collected data and formulated a clinical question | | 1 | 2 | 3 | 4 | 5 |
| 402 | Searched relevant evidence from recent national guidelines and literature | | 1 | 2 | 3 | 4 | 5 |
| 403 | Read and critically appraised a clinical research study | | 1 | 2 | 3 | 4 | 5 |
| 404 | Applied findings of clinical literature into clinical practice | | 1 | 2 | 3 | 4 | 5 |
| 405 | Looked at recent national guidelines and new treatment protocols | | 1 | 2 | 3 | 4 | 5 |
| 406 | Share evidence from a study/ies in the  form of report or presentation to hospital staffs? | | 1 | 2 | 3 | 4 | 5 |
| 407 | Shared evidence from a study/ies in the  form of report or presentation to hospital staff | | 1 | 2 | 3 | 4 | 5 |
| 408 | Shared new national guidelines or treatment protocols with a colleague | | 1 | 2 | 3 | 4 | 5 |
| 409 | Changed existing clinical practice based on recent clinical studies | | 1 | 2 | 3 | 4 | 5 |
| 410 | Evaluated clinical practice based on recent studies | | 1 | 2 | 3 | 4 | 5 |
| 411 | Added new types of healthcare based on literature | | 1 | 2 | 3 | 4 | 5 |
| 412 | Discussed with patient/family member changing types of care | | 1 | 2 | 3 | 4 | 5 |
| 413 | Changed practice based on information you received from in-service training/conferences? | | 1 | 2 | 3 | 4 | 5 |
| 414 | Evaluated patient outcomes after practice change | | 1 | 2 | 3 | 4 | 5 |
| 415 | Accessed and read health-related national policies and regulations | | 1 | 2 | 3 | 4 | 5 |
| 416 | Accessed the following websites to answer clinical question? | 1 | 2 | 3 | 4 | 5 | 4 |
|  |  | 1 | 2 | 3 | 4 | 5 | 4 |
|  |  | 1 | 2 | 3 | 4 | 5 | 4 |
|  |  | 1 | 2 | 3 | 4 | 5 | 4 |
|  |  | 1 | 2 | 3 | 4 | 5 | 4 |

**Part V: Competence**

Assessing Competency in Evidence Based Medicine (ACE tool): Read through the following information on patient scenario, clinical question, search strategy and article extract before answering the following set of questions and then respond as ‘**yes’** or ‘**no’** . (Please see the case scenario of patient given before answering the next questions).

| S.N o | **Questions** | Yes(1) | No  (2) |
| --- | --- | --- | --- |
| **Asking an answerable question** | | | |
| 501 | Are all PICO elements described in the patient scenario? | 1 | 2 |
| 502 | Does the question constructed post-scenario provide a focused,  Foreground question? | 1 | 2 |
| **Searching the literature** | | | |
| 503 | Will the search strategy (to be used in Medline) retrieve relevant studies relating to the question? | 1 | 2 |
| 504 | Does the search strategy utilize appropriate MeSH/keywords and  Boolean operators correctly and effectively? | 1 | 2 |
| **Appraising the evidence** | | | |
| 505 | Was there sufficient information to determine the representativeness of the study participants? | 1 | 2 |
| 506 | Was the method of participant allocation to intervention/exposure and comparison adequate? | 1 | 2 |
| 507 | Was any form of adjustment required? | 1 | 2 |
| 508 | Were all participants blinded to the treatment/exposure? | 1 | 2 |
| 509 | Were all investigators blinded to the treatment/exposure? | 1 | 2 |
| 510 | Were all outcome assessors blinded to the treatment/exposure? | 1 | 2 |
| 511 | Were all patients analyzed in the groups to which they were randomized? | 1 | 2 |
| **Applying the evidence** | | | |
| 512 | Does the patient in the scenario share similar characteristics/circumstances to participants in the study? | 1 | 2 |
| 513 | Is the treatment/therapy feasible in the clinical setting of the scenario? | 1 | 2 |
| 514 | Were all clinically important outcomes considered? | 1 | 2 |
| 515 | Do the likely benefits of the treatment/therapy outweigh any potential harms and costs? | 1 | 2 |

Thank you for your time!!!
